# Supplementary material for: Endothelial Cell Dysfunction and Hypoxia as Potential Mediators of Pain in Fabry Disease: A Human-Murine Translational Approach
Source: Int J Mol Sci. 2023 Oct 21;24(20):15422. doi: 10.3390/ijms242015422 (PMC10607880; doi:10.3390/ijms242015422)
Supplement: Supplementary file 1 [file ijms-24-15422-s001.zip › ijms-2629678-supplementary.pdf]

International Journal of
Molecular Sciences

MDPI

Supplement

Supplementary Table S1. Human primary and secondary antibodies for immunolabeling of human EC.

**Primary Antibody**	**Species**	**Dilution**	**Company**
HIF1a	Goat anti-human	1:100	R&D Systems, Minneapolis, MN, USA
HIF2	Rabbit anti-human	1:100	Bio Techne, Abingdon, UK
CD31/PECAM1	Mouse anti-human	1:100	Abcam, Cambridge, UK
TOMM20	Moise anti-human	1:200	Santa Cruz Biotechnology, Santa Cruz, CA, USA
VE-Cadherin	Rabbit anti-human	1:200	Abcam, Cambridge, UK
VEGFA	Goat anti-human	1:100	R&D Systems, Minneapolis, MN, USA
Secondary Antibody	Species	Dilution	Company
AlexaFluor® 647	Donkey anti-rabbit IgG (H + L)	1:400	Jackson ImmunoResearch Laboratories Inc., West Grove, PA, USA
Cy3	Donkey anti-goat IgG (H + L)	1:100	Jackson ImmunoResearch Laboratories Inc., West Grove, PA, USA
Cy3	Donkey anti-mouse IgG (H + L)	1:100	Jackson ImmunoResearch Laboratories Inc., West Grove, PA, USA
Cy3	Donkey anti-rat IgG (H + L)	1:400	Jackson ImmunoResearch Laboratories Inc., West Grove, PA, USA
DAPI	---	1:10 000	Sigma-Aldrich, St. Louis, MS, USA

Abbreviations: CD31: cluster of differentiation 31, Cy3: cyan 3, DAPI: 4′,6-diamidino-2-phenylindole, HIF1a/2: hypoxia-inducible factor 1a/2, PECAM1: platelet endothelial cell adhesion molecule, TOMM20: translocase of outer mitochondrial membrane 20, VE-Cadherin: vascular endothelial cadherin, VEGF: vascular endothelial growth factor A.

Supplementary Table S2. Hypoxia-associated gene expression assays for duplex qRT PCR of human EC.

Target gene	Source	Assay ID
ADM	Thermo Fisher Scientific (Waltham, MA, USA)	Hs00969450_g1
CAV1		Hs00971716_m1
ENG		Hs00923996_m1
EPAS1 (HIF2a)		Hs01026149_m1
GAPDH (endogenous control)		Hs02786624_g1
GLA		Hs00609238_m1
TGFb1		Hs00998133_m1
TNF		Hs00174128_m1
VEGF		Hs00900055_m1

Abbreviations: *ADM*: adrenomedullin, *CAV1:* caveolin 1, *ENG*: endoglin, *EPAS1*: endothelial PAS domain protein 1, *EPO*: erythropoietin, *GAPDH*: glyceraldehyde 3-phosphate dehydrogenase, *HIF2a*: hypoxia-inducible factor 2a, *LRG1*: leucin-rich alpha-2-glycoprotein 1, *TGFβ1*: transforming growth factor beta 1, *TNF*: tumor necrosis factor, *VEGF*: vascular endothelial growth factor.

Supplementary Table S3. Murine primary and secondary antibodies for immunolabeling of murine DRG.

**Primary Antibody**	**Species**	**Dilution**	**Company**
CA9	Rabbit anti-mouse	1:100	Invitrogen, Carlsbad, CA, USA
CD31/PECAM1	Rat anti-mouse	1:100	Bio-Rad Laboratories Inc., Hercules, CA, USA
HIF1a	Goat anti-mouse	1:100	R&D Systems, Minneapolis, MN, USA
PGP9.5	Guinea pig anti-mouse	1:100	Sigma-Aldrich, St. Louis, MS, USA
**Secondary Antibody**	**Species**	**Dilution**	**Company**
AlexaFluor® 488	Donkey anti-guinea pig IgG (H + L)	1:400	Jackson ImmunoResearch Laboratories Inc., West Grove, PA, USA
AlexaFluor® 647	Donkey anti-rabbit IgG (H + L)	1:400	Jackson ImmunoResearch Laboratories Inc., West Grove, PA, USA
Cy3	Donkey anti-goat IgG (H + L)	1:400	Jackson ImmunoResearch Laboratories Inc., West Grove, PA, USA
Cy3	Donkey anti-rat IgG (H + L)	1:400	Jackson ImmunoResearch Laboratories Inc., West Grove, PA, USA
DAPI	---	1:10 000	Sigma-Aldrich, St. Louis, MS, USA

Abbreviations: CA9: carbonic anhydrase 9, CD31: cluster of differentiation 31, Cy3: Cyan 3, DAPI: 4′,6-diamidino-2-phenylindole, HIF1a: hypoxia-inducible factor 1a, PECAM1: platelet endothelial cell adhesion molecule, PGP9.5: protein gene product 9.5.

Supplementary Table S4. Hypoxia-associated gene expression assays for duplex qRT PCR of murine DRG tissue.

Target gene	Source	Assay ID
BNIP3	Thermo Fisher Scientific (Waltham, MA, USA)	Mm01275600_g1
CA9		Mm01349478_m1
DDIT4		Mm00512504_g1
EGLN3		Mm00472200_m1
EPO		Mm00433126_m1
FOS		Mm00487425_m1
HIF1a		Mm00468869_m1
HK2		Mm00443385_m1
LDHA		Mm01612132_g1
LGALS3		Mm00802901_m1
MMP2		Mm00439498_m1
MMP3		Mm00440295_m1
MMP9		Mm00442991_m1
PDK1		Mm0054300_m1
RPL13a (endogenous control)		Mm01612986_gH
SLC2A1 (GLUT1)		Mm00441480_m1
TFRC		Mm00441941_m1

Abbreviations: *BNIP3*: BCL2 interacting protein 3, *CA9*: carbonic anhydrase 9, *DDIT4*: DNA damage-inducible factor 4, *EGLN3*: Egl-9 family hypoxia-inducible factor 3, *EPO*: erythropoietin, *FOS*: FBJ osteosarcoma oncogene, *GLUT1*: glucose transporter 1, *HIF1a*: hypoxia-inducible factor 1a, *HK2*: hexokinase 2, *LDHA*: lactate dehydrogenase A, *LGALS3*: lectin galactose binding soluble 3, *MMP2/3/9*: matrix metalloproteinase 2/3/9, *PDK1*: phosphoinositide-dependent kinase 1, *RPL13a*: ribosomal protein L13a, *SLC2A1*: solute carrier family 2, *TFRC*: transferrin receptor.
